# Heavy metal load and effects on biochemical properties in urban soils of a medium-sized city, Ancona, Italy

**DOI:** 10.1007/s10653-021-01105-8

**Published:** 2021-10-06

**Authors:** Dominique Serrani, Franco Ajmone-Marsan, Giuseppe Corti, Stefania Cocco, Valeria Cardelli, Paola Adamo

**Affiliations:** 1 Department of Agriculture, Food and Environmental Sciences, Polytechnic University of Marche, Via Brecce Bianche 10, 60131 Ancona, Italy; 2 Department of Agriculture, Forest and Food Sciences, University of Turin, Largo Paolo Braccini 2, 10095 Grugliasco, Italy; 3 Department of Agricultural Sciences, University of Naples Federico II, Via Università 100, 80055 Portici, Italy

**Keywords:** Urban soil pollution, Heavy metals, Microbial biomass C, Enzyme activities, Soil pollution benchmark

## Abstract

Urban soils are often mixed with extraneous materials and show a high spatial variability that determine great differences from their agricultural or natural counterparts. The soils of 18 localities of a medium-sized city (Ancona, Italy) were analysed for their main physicochemical and biological properties, and for chromium (Cr), copper (Cu), cobalt (Co), lead (Pb), nickel (Ni), zinc (Zn), and mercury (Hg) total content, distribution among particle-size fractions, and extractability. Because of the absence of thresholds defining a hot spot for heavy metal pollution in urban soils, we defined a “threshold of attention” (ToA) for each heavy metal aiming to bring out hot spot soils where it is more impellent to intervene to mitigate or avoid potential environmental concerns. In several city locations, the soil displayed sub-alkaline pH, large contents of clay-size particles, and higher TOC, total *N*, and available *P* with respect to the surrounding rural areas, joined with high contents of total heavy metals, but low availability. The C biomass, basal respiration, qCO_2_, and enzyme activities were compared to that detected in the near rural soils, and results suggested that heavy metals content has not substantially compromised the soil ecological services. We conclude that ToA can be considered as a valuable tool to highlight soil hot spots especially for cities with a long material history and, for a proper risk assessment in urban soils, we suggest considering the content of available heavy metals (rather than the total content) and soil functions.

**Supplementary Information:**

The online version contains supplementary material available at 10.1007/s10653-021-01105-8.

## Introduction

Urban soils are soils of urban and suburban environments intensively disturbed by human activity (Morel et al., 2005; Pouyat et al., 2020) and that show a remarkable spatial heterogeneity (De Kimpe et al. 2000). Historically, urban soils were ignored in soil studies, but in the last decades, they have gained attention because of their importance for human health in the city environment (Rossiter, 2007). In addition, the urban population was 34% of the total global population in 1960, amounted to 54% in 2014, and is expected to be 68% in 2050 (UN, 2018; WHO, 2016). Because of this, urban soil pollution may represent a global growing environmental problem (FAO & ITPS 2015; Li et al., 2018).

Among the several types of pollutants, heavy metals are the most frequent ones in urban soils (Kumar & Hundal, 2016). According to numerous studies (e.g. Ajmone-Marsan & Biasioli, 2010; Sodango et al., 2018; Wuana & Okieimen, 2011; Xiao et al., 2017; Zhang & Whang 2020), the main sources of heavy metals in urban soil pollution are metallurgical industry, mining activities, fossil fuel consumption, vehicular traffic, irrigation, waste incineration, and, to a certain extent, the use of fertilizers and agrochemicals. The urban intensity and concentration of the emission sources are often higher than elsewhere, with a redistribution of heavy metals and other pollutants by wind and atmospheric deposition even at considerable distances from the source (Liao et al., 2020; Liu et al., 2016; Wei & Yang, 2010).

Because of the relationships between soil and the rest of the ecosystem, studies investigated the potential risk that heavy metals represent for human health in the urban environment (e.g. Ajmone-Marsan & Biasioli, 2010; Bugnot et al., 2019; Francová et al., 2016; Li et al., 2018; Luo et al., 2015). To assess their potential translocation to other environmental compartments, Ajmone-Marsan et al. (2008) investigated the distribution of potentially toxic heavy metals in soils of five European cities, while, in other cities, the distribution of potentially toxic heavy metals across the city or urban parks was assessed by Madrid et al. (2006), Simon et al. (2013) and Pons-Branchu (2015). In China, heavy metals pollution and translocation has become a serious environmental problem with the intense industrialization and urbanization of the last two decades, and the situation is monitored all over the country (e.g. Cheng et al., 2014; Hu et al., 2020; Li et al., 2014, 2017; Luo et al., 2012; Tepanosyan et al., 2016; Yuan et al., 2014, 2021). However, point sources may have a harsh impact, but it is the diffuse heavy metal pollution that causes the most severe problems to the soil biochemical and microbiological properties (e.g. Lorenz & Kandeler, 2005; Naylo et al., 2019; Papa et al., 2010; Unda-Calvo et al., 2019).

Because of environmental and human health concern, many countries adopted regulatory guidelines defining the limits for soil metal(loid)s in soil. Examples are UK (Environment Agency, 2020), France (Darmendrail, 2003), China (Shi-bao et al., 2018), USA (United States Environmental Protection Agency, 2021), Russia and Netherland (Vodyanitskii, 2016), and Italy (Gazzetta Ufficiale No. 293, 15 December 1999). However, guidelines often do not differentiate urban soils from other types of soil. Instead, limits for heavy metals contaminating urban soils would be useful for cities with long time of human settlement where metallurgy, construction, and many other human activities have accumulated heavy metals in soil for centuries or millennia. Because of this, after a thorough evaluation of the microbial soil functionality of soils differently affected by heavy metal concentrations in a city with a long human history (Ancona, Italy), we propose the “threshold of attention”, a limit for heavy metals in urban soils that can change in time depending on the sources of pollution and the remediation measures activated.

Therefore, the present study aimed at: *i*) assessing the total and available concentrations of heavy metals (Co, Cr, Cu, Hg, Ni, Pb, and Zn) in the soils of a medium-sized city (Ancona, Italy); (*ii*) examining the effect of heavy metals on soil biochemical properties as proxies for soil functionality [microbial biomass C content, cumulative CO_2_ respiration, metabolic quotient, and the activities of four of the most active soil enzymes (alkaline phosphatase, acid phosphatase, ß-glucosidase, and urease)]; (*iii*) proposing a “threshold of attention” for heavy metal pollution in urban soils. The potential of this study approach is to better assess environmental risks related to the presence of heavy metals in the soils of old-settled cities.

## Materials and methods

### General characteristics of Ancona

**Fig. 1:**
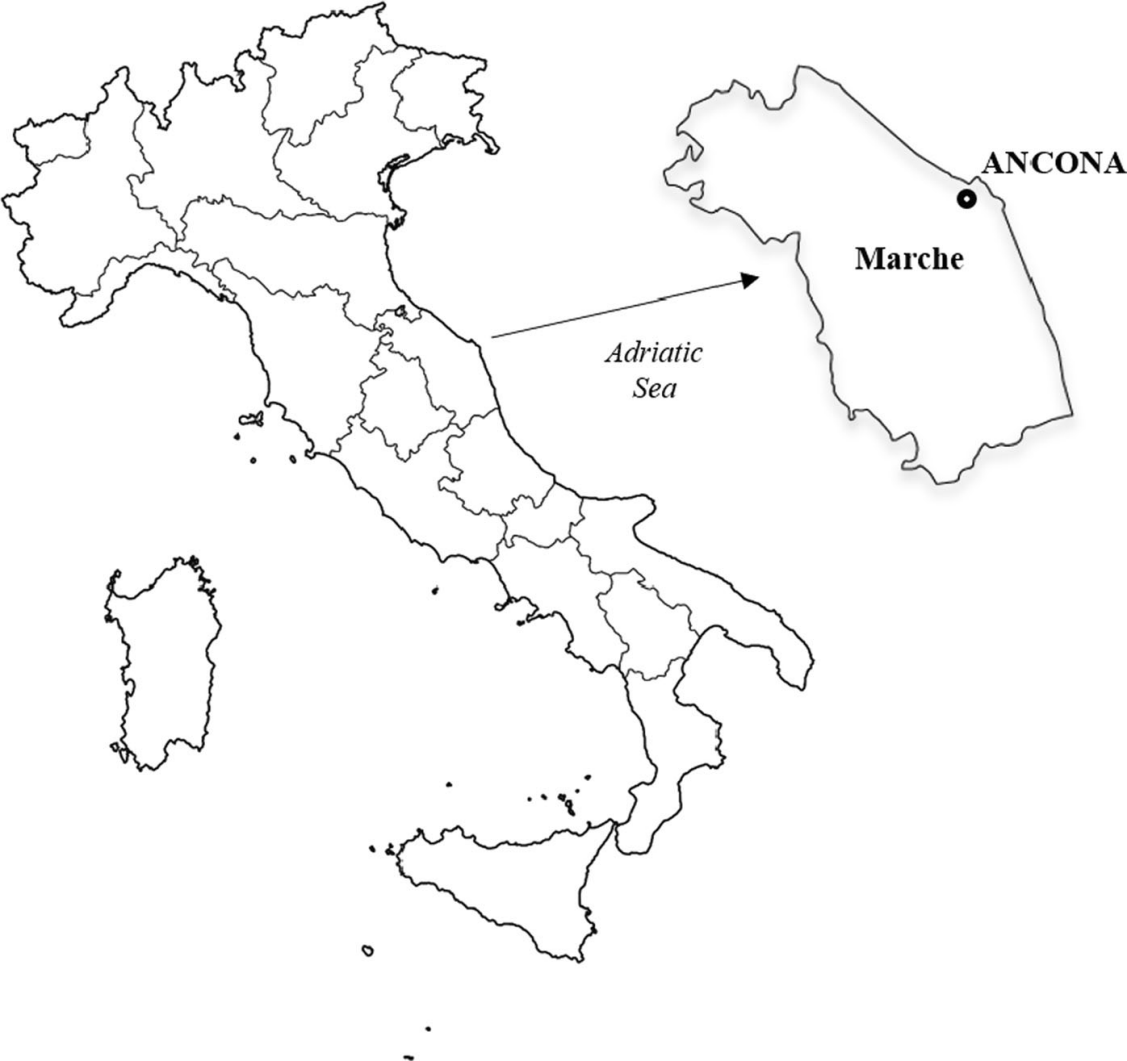
Map of Italy with localization of the Ancona City in Marche region

The city of Ancona is located on the eastern coast of central Italy (Fig. 1). Because of its strategic position on the Adriatic Sea, the area of the city has been populated since the Bronze Age. Nowadays, the Municipality of Ancona has a surface of ≈125 km^2^, a population of ≈100 300 inhabitants, and a population density of ≈803 inhabitants per km^2^. The city has railway and bus stations and hosts the main harbour of the Adriatic Sea and other touristic facilities that connect Italy to Balkan countries and Greece, with strong trade activities (fishery) that have largely influenced the economic and urban development. At ≈10 km from the city in the W-NW direction, there is an oil refinery (43°38′17.47"N, 13°22′45.44"E). In the last decade, technical investigations reported that the refinery has no considerable socio-environmental impact farther than 4–5 km from the plant (ARPAM, 2012; Corti et al., 2010). It is therefore sufficiently far to exclude any influence on the city.

The city has a typical sub-Mediterranean climate, with a mean annual air temperature of 13.6 °C, and a mean annual precipitation of 780 mm and receives dominant and strong winds from N and NW (Brecciaroli et al., 2012). The urban area is spread on fine-textured Plio-Pleistocene carbonaceous marine and fluvio-marine sediments, a lithologic unit present in the coastal and peri-coastal area of the Marche region (Cocco et al., 2007, 2013), forming a series of gentle hills with maximum altitudes of ≈140 m.

### Selected locations, soil collection, and soil processing

**Fig. 2:**
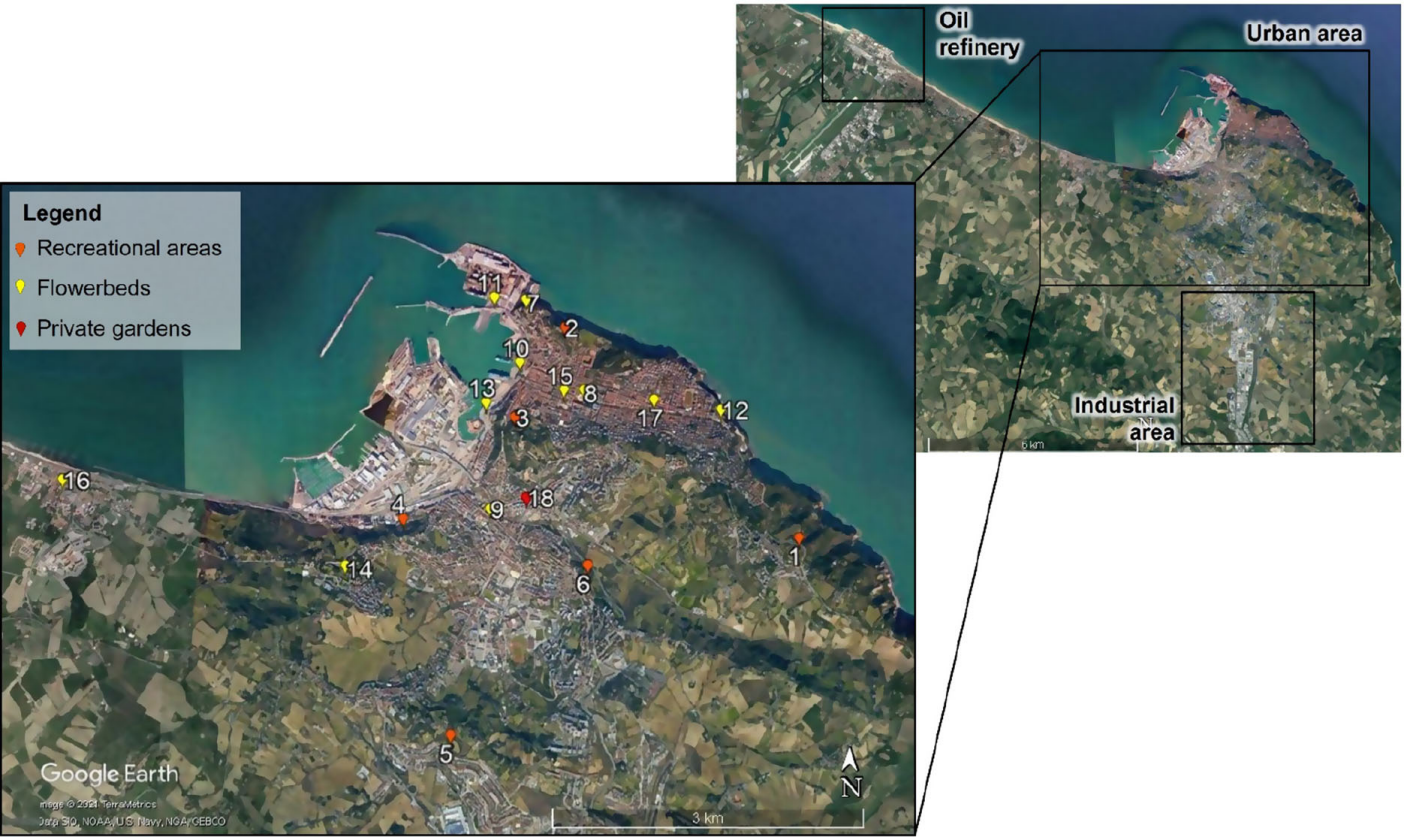
Sites localization of the urban soils collected at Ancona City (central Italy). Recreational areas: 1. Altavilla park; 2. Cardeto park; 3. Cittadella park; 4. Palombella park; 5. Unicef park; 6. Villa Beer park. Flowerbeds: 7. Cathedral; 8. Cavour square; 9. Corso Carlo Alberto (avenue); 10. Harbour, close to the customs house; 11. Harbour, close to the Trajan arch; 12. Passetto neighbourhood; 13. Porta Pia neighbourhood; 14. Posatora neighbourhood; 15. Stamira square; 16. Torrette neighbourhood; 17. Viale della Vittoria. Private gardens: 18. Corso Carlo Alberto.

This investigation was carried out on the ancient and more densely populated part of the city, where also harbour, railway, and bus station are settled, and where large gardens are few. Within the ancient city, industrial activities were present until the 1990s; since then, they have been moved to scarcely inhabited outlying districts. Hence, 18 locations among the most representative ones of the city were selected, taking into consideration three urban land uses: recreational areas (6 locations), flowerbeds (11 locations), and private gardens (1 location) (Fig. 2). The main physiographic characteristics of each location are reported in Table 1. We are aware of the great dimensional heterogeneity and spatial variability of the studied locations but, in selecting them, we wanted to cover most of the situations where the soil is present in the city, as also suggested by Pouyat et al., (2017, 2020) and Burghardt (2017). Further, our aim was not to assess the spatial variability of the soil within locations, but to estimate the level of heavy metal concentrations within the city. Further details on the selected locations are reported at point 1 of Supplementary Materials).

**Table 1:** Sampling sites with details about location, physiography, soil use, and vegetation of the soils from different locations of the city of Ancona (central Italy)

Location	Geographic coordinates	Extension	Slope, %	Vegetation	Observations
Recreational areas*					
Altavilla park	43°36′11.12''N 13°32′35.02''E	1.2 ha	< 0.5	*Populus alba* L., *Fraxinus ornus* L., and *Robinia pseudoacacia* L	
Cardeto park	43°37′20.84''N 13°30′53.03''E	15 ha	< 0.5	Mediterranean vegetation with *Robinia pseudoacacia* L. and *Ailanthus altissima* (Mill.) Swingle	
Cittadella park	43°36′50.70''N 13°30′30.93''E	14 ha	30	Mixed meadow with *Cupressus sempervirens* L. and *Pinus halepensis* Mill	
Palombella park	43°36′17.31''N 13°29′41.84''E	10 ha	30	Rarefied grass; close to a dismissed brick factory	
Unicef park	43°35′07.74''N 13°30′02.96''E	6 ha	< 0.5	Mixed meadow with *Quercus ilex* L. and *Laurus nobilis* L	
Villa Beer park	43°36′02.15''N 13°31′02.53''E	5 ha	< 0.5	Mixed meadow with *Fraxinus ornus* L., *Pinus halepensis* Mill., *Quercus ilex* L., and *Viburnum tinus* L	
*Flowerbeds*					
Cathedral	43°37′30.04''N 13°30′35.85''E	330 m^2^	< 0.5	Mixed meadow with *Pittosporum tobira* (Thunb.) W.T. Aiton and *Robinia pseudoacacia* L	
Cavour square	43°36′59.85''N 13°31′01.25''E	1.1 ha	< 0.5	Rarefied grass with *Phoenis canariensis* Chabaud and *Tilia platyphyllos* Scop	
Corso Carlo Alberto (avenue)	43°36′20.43''N 13°30′19.90''E	1500 m^2^ (2.5 m wide, 600 m long)	< 0.5	*Pittosporum tobira* (Thunb.) W.T. Aiton and *Platanus occidentalis* L	Very congested area
Harbour, close to the customs house	43°37′09.10''N 13°30′33.03''E	89 m^2^	20	*Pittosporum tobira* (Thunb.) W.T. Aiton	Very congested area
Harbour, close to the Trajan arch	43°37′30.96''N 13°30′21.76''E	368 m^2^	< 0.5	Mixed meadow	
Passetto neighbourhood	43°36′53.28''N 13°32′01.80''E	289 m^2^	< 0.5	Mixed meadow with *Quercus ilex* L. and *Laurus nobilis* L	
Porta Pia neighbourhood	43°36′55.79''N 13°30′18.12''E	472 m^2^	< 0.5	Mixed meadow, *Pittosporum tobira* (Thunb.) W.T. Aiton and *Phoenis canariensis* Chabaud	
Posatora neighbourhood	43°36′02.01''N 13°29′16.66''E	300 m^2^	< 0.5	Mixed meadow with *Pinus pinea* L., *Nerium oleander* L., *Buddleja davidii* Franch., *Pittosporum tobira* (Thunb.) W.T. Aiton, and *Laurus nobilis* L	
Stamira square	43°36′59.92''N 13°30′52.51''E	0.12 ha	< 0.5	Mixed meadow with *Pittosporum tobira* (Thunb.) W.T. Aiton	
Torrette neighbourhood	43°36′30.04''N 13°27′11.99''E	0.19 ha	5	Mixed meadow with *Aesculus hippocastanum* L	Very congested area
Viale della Vittoria	43°36′56.59''N 13°31′32.29''E	2000 m^2^ (2 m wide, 1000 m long)	< 0.5	Mixed meadow with *Ulmus minor* Mill	Very congested area. Soil was replaced two years before the sampling
*Private gardens*					
Corso Carlo Alberto	43°36′24.22''N 13°30′35.85''E	190 m^2^	< 0.5	Vegetable garden and mixed meadow with sparse roses (*Rosa* spp.) and hedges of *Buxus sempervirens* L	Vegetable gardens, flowers, bushes

^*^The extension of recreational areas refers to the total park surface within which soil samples were collected in the surrounding of children’s games

At each location, three sampling sites were identified at the vertex of a triangle with sides of 6–8 m. Concerning with the depth of sampling, we collected the soil samples from the superficial 5 cm of soil with the purpose to collect the soil portion most enriched of heavy metals derived from allochthonous additions, as reported by Ljung et al. (2006) and Sun (2010). Therefore, from each site, a soil sample was taken by collecting the soil inside a 30 × 15 cm frame to the depth of 5 cm (≈2 kg). The three samples collected from each location were kept separately. Once in the laboratory, half of each sample at field moisture conditions was sieved through 2 mm to separate the skeletal fraction and maintained at field moisture at 4 °C for maximum one week before biochemical analyses. The other half of each sample was air-dried and sieved at 2 mm. All analyses were run on the < 2 mm fraction. During sample sieving, allochthonous materials like building debris, plastic, or scrap metal have never been found.

### Physicochemical analyses

The pH was determined potentiometrically in water at a 1:2.5 solid:liquid (w:v) ratio. Particle-size distribution was determined after the dissolution of organic cements with NaClO at pH 9 (Lavkulich & Wiens, 1970); sand was recovered by sieving at 0.05 mm, while silt was separated from clay by sedimentation. The content of active carbonate was estimated by the KMnO_4_ method (Drouineau, 1942), while that of total carbonates was measured using the gas volumetric method (Balázs et al., 2005; ISO, 10693, 1995). The content of total organic C (TOC) was estimated by wet digestion (Nelson & Sommers, 1996), and the total N was determined by a dry combustion analyser (Carlo Erba EA1110). Available *P* was estimated according to Olsen et al. (1954). The exchangeable cations were determined with a 0.2 M BaCl_2_ solution at pH 8.1 (solid:liquid ratio of 1:10) and the solution was analysed with flame mode for Ca, Mg, K, and Na by atomic absorption spectrophotometry (model AA-6300, Shimadzu, Germany).

The mineralogical assemblage was evaluated by X-ray diffractometry on manually compressed powdered samples. The diffractometer was a Philips PW 1830, which produced a Fe-filtered Co Kα1 radiation operating at 35 kV and 25 mA. After identification of the minerals on the basis of their characteristic peaks, a semi-quantitative assessment of the mineralogical composition was obtained by estimating the area of the peak by multiplying the peak height by its width at half-height. Calcite content was measured by dissolution (Bundy & Bremner, 1972). To determine the Cr and Ni contents of serpentine minerals, soil samples were gently fragmented and sieved at 1 mm to collect the 1–2 mm fraction. By a magnifying lens, from this fraction we separated aliquots of 100 to 300 mg rich of serpentine mineral. These aliquots were washed with diluted (0.25 M) HCl solution, rinsed with distilled water, ground, and analysed by X-ray diffraction; the aliquots with an estimated serpentine content larger than 90% were retrieved and treated with *aqua regia* (see below) to determine the lattice content of Cr and Ni.

For each soil sample, the pseudo-total concentration of Cr, Cu, Co, Pb, Ni, Zn, and Hg was obtained by dissolution of the specimens in *aqua regia* according to the following protocol: subsamples of 0.5 g were finely ground in an agate mortar and placed into a 120-mL Teflon-PFA microwave digestion vessel, added of 12 mL of *aqua regia* (1:24 soil:solution ratio) and digested at 0.69 × 10^6^ Pa for 10 min. The solution was then filtered through a Whatman 42 filter, transferred to 100-mL volumetric flasks, and brought to volume with distilled water. The contents of heavy metals were determined in the solution by inductively coupled plasma mass spectrometry (ICP-MS, Agilent 7500ce, Waldbronn, Germany). The same protocol was used to determine *i*) the pseudo-total concentrations of Co, Cr, Cu, Hg, Ni, Pb, and Zn in clay, silt, and sand separates (< 2, 2–50, and 50–2000 µm fractions, respectively) obtained by wet sieving and sedimentation of samples submerged in water for 2 h (no cement dissolution); and *ii*) the Cr and Ni contents of serpentine minerals obtained as mentioned above (in this case, we used aliquots of 100 mg). The detection limits for the considered elements were: 0.2 µg kg^−1^ for Co, Cr, Cu, and Pb; 0.4 µg kg^−1^ for Hg and Zn; 0.8 µg kg^−1^ for Ni.

The extractable (available) amounts of Cr, Cu, Ni, Pb, and Zn were estimated by 0.05 mol L^−1^ EDTA extraction at pH 7, with a 1:10 soil:solution ratio (Quevauviller et al., 1997) and the solutions were analysed by ICP-MS, Agilent 7500ce, Waldbronn, Germany. Extractable Co and Hg were not determined since a series of tests made on several samples gave always results below the respective detection limits.

### Biochemical analyses

The microbial biomass C (C_mic_) content was determined by the fumigation-extraction method of Vance et al. (1987), after 21 days of incubation at 25 °C and at 50% of the sample total water holding capacity. During this incubation period, basal respiration was obtained by measuring the respired CO_2_ by gas-chromatography (Blackmer & Bremner, 1977). Basal respiration was expressed as the cumulative amount of CO_2_–C evolved during the incubation period (∑CO_2_–C). The specific microbial respiration, or metabolic quotient (qCO_2_), expresses the CO_2_–C evolved per unit of microbial biomass C and time (µg CO_2_–C mg^−1^ C_mic_ h^−1^) and was calculated according to Anderson & Domsch (1993).

Alkaline and acid phosphatases activities were assayed according to Tabatabai (1994). Briefly, 1.00 g subsample at field moisture conditions was placed in a 50-ml flask and added of 0.2 ml of toluene, 4 ml of Modified Universal Buffer solution (MUB) at pH 6.5 for the assay of acid phosphatase or at pH 11 for the assay of alkaline phosphatase, and 1 ml of 0.05 M *p*-nitrophenyl phosphate solution. Once mixed the content, the stopped flask was placed in incubator at 37 °C. After 1 h, 1 ml of 0.5 M CaCl_2_ solution and 4 ml of 0.5 M NaOH solution were added, and the content mixed for few seconds. The soil suspension was filtered through a Whatman 42 filter, and the yellow colour intensity of the filtrate measured against a control at 420 nm by a Varian Cary® 50 UV–Vis spectrophotometer (Victoria, Australia). The ß-glucosidase activity was assessed by Eivazi & Tabatabai (1988), using the analogue substrate para-nitrophenyl-ß-D-glucopyranoside (*p*-NPG). A subsample of 1.00 g at field moisture conditions was put into a screw-cap glass tube (three replicates per sample) and incubated for 1 h in a water bath at 37 °C with 4 ml of 0.05 M MUB at pH 6.0 and 1 ml of 10 mM *p*-NPG solution dissolved in the buffer. The reaction was stopped by adding 1 ml of 0.5 M CaCl_2_ solution and 4 ml of 0.2 M tris(hydroxymethyl)aminomethane (tris) solution adjusted to pH 12 with NaOH. The mixture was centrifuged for 10 min at 1500 g and the absorbance measured at 410 nm by the Varian Cary® 50 UV–Vis spectrophotometer. Values were corrected for a blank (substrate added immediately after the addition of CaCl_2_ and tris–NaOH) and for the adsorption of para-nitrophenol (*p*-NP) released in the soil (Vuorinen, 1993). Urease activity was assessed per Tabatabai (1994). A subsample of 2.00 g at field moisture conditions was put into a 50-mL flask (three replicates) and incubated for 2 h in a water bath at 37 °C with 0.2 mL of toluene solution, 4 mL of 0.05 M tris solution adjusted to pH 9 with H_2_SO_4_, and 0.5 mL of 0.2 M urea solution. The reaction was stopped by adding 35 mL of 2.5 M KCl plus 0.32 mM Ag_2_SO_4_ solution. Once the suspension was cooled, it was brought to 50 mL with the KCl–Ag_2_SO_4_ solution. The ammonium released was measured on 20-mL aliquot of the suspension with an ammonium selective electrode after the addition of 0.1 mL of 10 M NaOH solution. A control without urea was measured for each sample (Tabatabai, 1982).

### Rationale for the proposed threshold of attention

As mentioned above, regulatory guidelines defining the soil limits for metal(loid)s often do not differentiate urban soils from other soil types, while limits for heavy metals contaminating urban soils would be useful especially for cities with a history of centuries or millennia during which human activities have introduced heavy metals in soil. Because of this, we propose the “threshold of attention” calculated as follows

$${\text{ToA }} = M_{a} + {\text{ }}\raise.5ex\hbox{$\scriptstyle 1$}\kern-.1em/ \kern-.15em\lower.25ex\hbox{$\scriptstyle 2$} SD$$

where

ToA = threshold of attention for each element;

*M*_a_ = arithmetic mean of the total concentrations;

*SD* = standard deviation.

Both *M*_a_ and SD were calculated at the city level and the choice to consider a half of the *SD* (½*SD*) was guided by the wish to bring out the hot spot soils where it is more impellent to intervene in order to mitigate or avoid potential environmental concerns. Practically, this threshold is mobile in time, meaning that, after recovery measures activated in some hot spots to reduce the heavy metal content, ToA may change and soils once under the threshold may then exceed it and require mitigating interventions.

### Data analysis

The physicochemical analyses were run in duplicate and the analytical mean was calculated. For each parameter, the analytical means of the three samples collected from each area were averaged to obtain the arithmetic mean and the standard deviation for each location (*n* = 3). The total concentrations of Co, Cr, Cu, Hg, Ni, Pb, and Zn in clay, silt, and sand separates, the extractable Cr, Cu, Ni, Pb, and Zn, and all the biochemical analyses were run in duplicate on one of the three samples collected per location; for these analyses, we report the analytical mean of the sample.

## Results

### Soil physicochemical properties

The pH values of the urban soils of Ancona ranged from 7.1 to 7.6, with an average value of ≈7.4 (Table 2). In general, the particle-size distribution ranged from silty clay loam to clay loam, with a few soils showing a silty loam texture (Table 2). The active carbonate represents the finest and more reactive carbonaceous particles; its content ranged from 40 to 156 g kg^−1^ with an average value of ≈109 g kg^−1^, which was roughly one-third of the total carbonates (Table 2). One exception was the flowerbed soil of Torrette neighbourhood, where values of active carbonate were low but represented ≈60% of the total carbonates. The contents of *TOC* (from 20.7 to 51.5 g kg^−1^) and total N (from 0.9 to 5.9 g kg^−1^) gave C/N ratios that, in 12 out of 18 locations, fell in the range 10–15 (Table 2). In the flowerbeds of the customs house of the Harbour and of Porta Pia, we obtained the lowest C/N ratio values (around 9), while the highest value was 48 at the Palombella park. Available *P* ranged from 6 to 202 mg kg^−1^ (Table 2), with an average value of 56 mg kg^−1^ and in 13 of the 18 locations the values were above the shortage limit of the method applied (23 mg kg^−1^). The content of exchangeable cations followed the order Ca >  > Mg > K ≥ Na, typical of soils derived from calcareous parent materials (Table 2).

**Table 2:** Main physicochemical properties for the soils from different locations of the city of Ancona (central Italy) and their general mean and median

	pH	Particle-size distribution			Total CaCO_3_	Active carbonate	Total organic C	Total N	Available P	Exchangeable cations			
		Sand	Silt	Clay						Ca	Mg	K	Na
		%			g kg^−1^				mg kg^−1^	cmol(+) kg^−1^			
*Recreational areas*													
Altavilla park	7.22(0.10)	18(2)	50(5)	32(3)	410.0(31.6)	141.5(10.3)	38.5(5.6)	3.2(0.7)	23.0(3.4)	27.5(1.4)	2.9(0.2)	2.0(0.4)	0.4(0.1)
Cardeto park	7.25(0.13)	42(22)	34(12)	24(10)	436.7(28.7)	130.3(24.7)	42.9(10.7)	3.6(1.6)	11.0(6.6)	23.2(1.0)	1.5(0.2)	1.0(0.0)	0.4(0.1)
Cittadella park	7.53(0.06)	9(2)	58(3)	33(5)	396.7(19.5)	133.3(3.9)	25.9(4.9)	2.4(0.8)	35.5(7.4)	19.9(0.8)	3.1(0.2)	1.3(0.1)	1.6(0.4)
Palombella park	7.57(0.12)	65(1)	22(1)	13(0)	741.1(17.9)	103.7(0.3)	43.5(5.9)	0.9(0.1)	11.1(1.6)	9.9(0.4)	0.8(0.1)	0.7(0.1)	0.6(0.2)
Unicef park	7.27(0.11)	16(2)	53(6)	31(4)	362.4(15.7)	128.2(3.4)	30.5(3.6)	2.4(0.4)	34.0(2.7)	26.9(1.3)	1.7(0.2)	1.2(0.2)	0.4(0.1)
Villa Beer park	7.49(0.07)	18(5)	48(5)	34(9)	263.3(13.8)	102.1(2.3)	24.8(2.9)	1.9(0.2)	6.0(1.1)	21.7(1.0)	2.8(0.2)	0.8(0.1)	0.4(0.1)
*Flowerbeds*													
Cathedral	7.36(0.07)	44(14)	40(4)	16(10)	496.7(25.7)	155.7(11.7)	36.6(2.4)	3.1(0.3)	113.0(9.6)	30.5(1.5)	1.7(0.1)	1.4(0.1)	0.4(0.0)
Cavour square	7.33(0.03)	24(5)	51(4)	25(2)	420.0(24.3)	146.7(0.7)	35.8(1.5)	3.0(0.1)	81.5(6.8)	27.0(0.9)	1.7(0.1)	0.7(0.0)	0.4(0.0)
Corso Carlo Alberto (avenue)	7.43(0.08)	42(11)	38(3)	20(8)	306.7(15.2)	119.7(9.3)	26.0(3.6)	2.1(0.8)	59.0(5.9)	22.8(0.9)	1.5(0.1)	1.4(0.2)	0.7(0.2)
Harbour, close to the customs house	7.30(0.07)	50(13)	31(8)	19(5)	421.3(20.9)	99.3(12.7)	32.4(7.5)	3.5(0.5)	141.3(12.9)	16.1(0.7)	1.0(0.0)	1.4(0.2)	0.5(0.1)
Harbour, close to the Trajan arch	7.54(0.08)	47(19)	35(10)	18(8)	252.2(17.5)	64.0(11.8)	30.6(5.4)	1.6(0.2)	37.0(2.8)	15.4(0.7)	1.7(0.1)	1.1(0.2)	0.5(0.0)
Passetto neighbourhood	7.31(0.11)	22(7)	53(7)	25(3)	283.3(7.6)	120.3(23.6)	47.9(5.7)	3.3(0.4)	32.0(3.3)	25.1(1.2)	3.4(0.2)	1.3(0.1)	1.4(0.3)
Porta Pia neighbourhood	7.46(0.15)	20(13)	51(5)	29(8)	240.5(8.3)	117.7(1.9)	51.5(18.6)	5.9(0.5)	43.0(3.4)	24.8(1.3)	2.8(0.3)	0.7(0.0)	0.5(0.2)
Posatora neighbourhood	7.43(0.10)	15(3)	47(4)	38(6)	356.7(18.0)	125.8(10.4)	30.3(2.2)	2.4(0.2)	17.0(3.0)	24.0(0.8)	2.8(0.0)	1.2(0.1)	0.4(0.0)
Stamira square	7.35(0.10)	16(6)	57(4)	27(3)	343.3(18.8)	128.3(1.8)	33.8(5.7)	1.8(0.2)	46.4(8.0)	23.0(0.6)	3.5(0.3)	1.1(0.0)	1.8(0.5)
Torrette neighbourhood	7.41(0.09)	13(1)	51(2)	36(2)	73.3(8.4)	42.7(12.2)	20.7(1.6)	1.4(0.2)	21.4(7.7)	22.5(0.8)	2.5(0.2)	0.8(0.1)	0.4(0.1)
Viale della Vittoria	7.21(0.08)	25(7)	49(4)	26(3)	150.2(10.1)	66.2(3.4)	25.5(3.4)	2.1(0.4)	93.0(13.1)	23.7(1.2)	2.4(0.2)	1.3(0.0)	0.6(0.0)
*Private gardens*													
Corso Carlo Alberto	7.09(0.07)	21(8)	49(4)	30(4)	100.0(13.2)	40.3(2.2)	41.8(7.4)	3.7(0.6)	202.0(18.5)	22.7(1.1)	1.7(0.2)	1.6(0.2)	0.7(0.1)
General mean	7.36(0.13)	28(16)	45(10)	27(7)	336.4(155.1)	109.2(34.5)	34.4(8.6)	2.7(1.1)	56.0(52.2)	22.6(4.9)	2.2(0.8)	1.2(0.3)	0.7(0.4)
Median	7.36	22	49	27	350.0	120.0	33.1	2.4	36.3	23.1	2.1	1.2	0.5

Numbers in parentheses are the standard deviations (for single locations, *n* = 3; for general mean, *n* = 18)

Many soils showed a mineralogical composition with calcite as the most represented mineral, followed by quartz, clay minerals, plagioclases, micas, kaolinite, and dolomite (Table 3). In some soils, small amounts of amphiboles, pyroxenes, primary chlorite, and serpentine (mainly antigorite) were also detected. The antigorite of these soils contained from 432 to 788 mg kg^−1^ Cr, and from 547 to 850 mg kg^−1^ Ni. Even though we cannot exclude the possibility that allochthonous materials were added to these soils, it is also true that antigorite is ubiquitous in amounts of 2–4% in a soil belt of the southern Po river delta that spans from the Adriatic coast to 10–15 km inland, often accompanied by small amounts of amphiboles and chlorite (Cocco et al., 2007). Instead, pyroxenes have rarely been detected in the same soils of this inland belt, but they were observed in three flowerbeds: the two at the harbour and one from Viale della Vittoria.

**Table 3:** Mineralogical composition (semi-quantitative estimation) for the soils from different locations of the city of Ancona (central Italy) and their general mean and median

	Quartz	Calcite	Dolomite	Plagioclases	Micas	Kaolinite	2:1 clay minerals	Chlorite	Pyroxenes	Amphiboles	Serpentine
	(%)										
*Recreational areas*											
Altavilla park	12(3)	37(5)	11(3)	10(1)	9(2)	8(1)	13(3)	0(-)	0(-)	0(-)	tr
Cardeto park	16(5)	41(2)	9(4)	10(0)	11(2)	5(2)	8(0)	0(-)	0(-)	0(-)	tr
Cittadella park	12(4)	39(5)	8(3)	9(3)	10(0)	7(2)	15(3)	0(-)	0(-)	0(-)	0(-)
Palombella park	13(2)	54(4)	5(0)	9(2)	9(2)	9(2)	tr	0(-)	0(-)	0(-)	1(0)
Unicef park	15(3)	34(5)	8(2)	11(1)	9(2)	8(1)	15(2)	0(-)	0(-)	0(-)	0(-)
Villa Beer park	13(4)	27(6)	14(2)	12(0)	12(0)	10(0)	12(1)	0(-)	0(-)	0(-)	1(1)
*Flowerbeds*											
Cathedral	15(3)	48(6)	6(2)	11(0)	10(1)	9(0)	tr	0(-)	0(-)	0(-)	1(0)
Cavour square	14(7)	38(3)	4(6)	11(1)	12(4)	10(0)	11(2)	0(-)	0(-)	0(-)	0(-)
Corso Carlo Alberto (avenue)	23(5)	22(5)	0(-)	19(3)	14(3)	10(2)	12(2)	0(-)	0(-)	0(-)	0(-)
Harbour, close to the customs house	21(2)	32(9)	4(1)	11(11)	8(3)	8(2)	8(0)	0(-)	3(2)	5(0)	0(-)
Harbour, close to the Trajan arch	16(7)	24(3)	7(4)	19(4)	9(4)	6(4)	10(4)	0(-)	4(2)	2(0)	3(3)
Passetto neighbourhood	14(0)	25(8)	10(0)	12(7)	11(2)	8(3)	7(4)	2(0)	0(-)	5(0)	6(0)
Porta Pia neighbourhood	14(5)	30(5)	14(10)	10(1)	10(3)	4(0)	11(0)	0(-)	0(-)	7(0)	tr
Posatora neighbourhood	14(2)	30(5)	9(1)	13(2)	11(2)	8(2)	14(2)	0(-)	0(-)	0(-)	1(0)
Stamira square	17(6)	31(2)	7(0)	9(3)	12(4)	6(0)	11(3)	0(-)	0(-)	7(0)	tr
Torrette neighbourhood	20(4)	21(1)	7(4)	15(3)	12(4)	8(2)	12(2)	0(-)	0(-)	0(-)	5(0)
Viale della Vittoria	18(3)	24(4)	0(-)	13(1)	12(1)	10(2)	12(1)	0(-)	6(2)	5(1)	tr
*Private gardens*											
Corso Carlo Alberto	18(4)	30(5)	0(-)	10(0)	13(2)	10(1)	12(1)	0(-)	0(-)	4(1)	3(1)
General mean	16(3)	33(9)	7(4)	12(3)	11(2)	8(2)	10(4)	0(0)	1(2)	2(3)	2(2)
Median	15	31	7	11	11	8	12	0	0	0	1

Numbers in parentheses are the standard deviations (for single locations, *n* = 3; for general mean, *n* = 18)

### Heavy metals in the city soils

Over the 18 locations, five out of the seven elements considered (Co, Cr, Cu, Hg, and Ni) showed ToA similar or lower than the limits of the Italian legislation, while for Pb and Zn ToA was higher (Table 4). Each heavy metal exceeded its ToA in three to five locations. For example, Cu and Zn showed concentrations higher than their ToA in three locations, Co overcame ToA in four locations, while Cr, Pb, Ni, and Hg exceeded their ToA in five locations. As a whole, in nine out of 18 locations ToAs were exceeded by two to six heavy metals. In details, the flowerbed close to the custom house at the Harbour was the worst location, with six metals above ToA (Cr, Cu, Hg, Ni, Pb, and Zn) and both Pb and Zn with the highest concentrations found in the city soils (317 and 592 mg kg^−1^, respectively). In four sites, three heavy metals exceeded their ToA: the exciding elements were Hg, Ni, and Pb in the Cavour square flowerbed; Co, Cr, and Ni in the Passetto and Posatora flowerbeds; Cu, Pb, and Zn in the Corso Carlo Alberto private gardens. In other four locations, two elements were higher than ToA: Cu and Zn in the recreational area of Palombella Park; Hg and Pb in the Cathedral and Porta Pia flowerbeds; Co and Ni in the flowerbed close to the Trajan arch at the Harbour. Finally, in four locations one heavy metal overcame ToA: Cr in the recreational area of Altavilla park and the flowerbed at Torrette, Hg in the recreational area of Cardeto park, and Co in the recreational area of Cittadella park.

**Table 4:** Total concentrations of heavy metals for the soils from different locations of the city of Ancona (central Italy) and their general mean, median, and threshold of attention

	Co	Cr	Cu	Hg	Ni	Pb	Zn
	mg kg^−1^						
*Recreational areas*							
**Altavilla park**	18(0)	**87(10)**	48(24)	0.11(0.05)*	49(7)	66(12)	187(29)**†**
**Cardeto park**	16(2)	28(8)	28(6)	0.86(0.23)*	45(12)	52(8)	105(12)
**Cittadella park**	**20(1)†**	39(4)*	37(13)	0.25(0.04)	50(10)	87(21)	124(38)*
**Palombella park**	15(2)	31(4)	282(38)*†	0.02(0.01)*	40(15)	89(11)	280(-)**†
Unicef park	19(2)	45(7)	33(8)	0.14(0.03)	53(27)*	42(9)	76(7)
Villa Beer park	16(2)	35(2)	21(13)	0.04(0.03)	43(17)	25(4)	67(25)
*Flowerbeds*							
**Cathedral**	16(0)	21(3)	41(8)	0.93(0.31)*	31(21)	192(48)*†	268(47)*†
**Cavour square**	19(1)	46(4)	56(2)	**0.50(0.19)**	**59(3)**	**173(7)†**	191(27)**†**
Corso Carlo Alberto (avenue)	17(1)	48(2)	57(46)	0.03(0.02)*	50(1)	70(65)	212(150)**†**
**Harbour, close to the customs house**	19(1)	**58(7)**	**141(24)†**	**0.88(0.81)**	**58(4)**	**317(51)†**	**592(124)†**
**Harbour, close to the Trajan arch**	23(2)*†	51(8)	36(-)**	0.22(0.09)*	**77(22)**	46(-)**	118(36)
**Passetto neighbourhood**	**22(0)†**	56(-)**	42(2)	0.16(0.04)	**58(17)**	62(15)*	176(15)*†
**Porta Pia neighbourhood**	19(1)	37(3)*	55(17)	**0.45(0.09)**	45(19)	221(68)*†	255(28)**†**
**Posatora neighbourhood**	**20(2)†**	**53(6)**	35(9)	0.05(0.03)*	**56(11)**	33(-)**	83(20)
Stamira square	18(2)	38(2)	38(4)	0.12(0.04)	53(2)	43(3)	141(28)
**Torrette neighbourhood**	17(1)	**58(5)**	31(1)	0.07(0.03)*	51(7)*	31(5)	75(5)
Viale della Vittoria	16(2)	46(13)	42(16)	0.06(0.03)	50(6)	46(11)	137(42)
*Private gardens*							
**Corso Carlo Alberto**	16(1)	44(8)	**127(18)†**	0.21(0.06)	48(8)	**159(49)†**	**496(159)†**
General mean	18.1(2.2)	45.6(14.6)	63.9(63.1)	0.28(0.31)	50.9(9.5)	97.4(81.3)	199.1(143.0)
Median	18.0	45.5	41.5	0.15	50.0	64.0	158.5
Proposed threshold of attention (general mean + ½ standard deviation)	19.2	52.9	95.5	0.44	55.7	138.1	270.6

Numbers in parentheses are the standard deviations (for single locations, *n* = 3; for general mean, *n* = 18). Locations in bold are those with one or more heavy metals overcoming the threshold of attention; the values in bold are those overcoming the threshold of attention for the respective heavy metal

*n = 2

**n = 1

^**†**^Concentrations exceeding the limit for residential areas according to the Italian law, which are: 20 mg kg^−1^ for Co; 150 mg kg^−1^ for Cr; 120 mg kg^−1^ for Cu; 1 mg kg^−1^ for Hg; 120 mg kg^−1^ for Ni; 100 mg kg^−1^ for Pb; 150 mg kg^−1^ for Zn (Gazzetta Ufficiale No. 293, 15 December 1999)

Table 5 shows the total content of the seven elements in each soil separate. In general, Cr, Cu, Hg, Ni, and Zn displayed the highest concentrations in the clay (or silt) fraction, while Pb often showed the highest concentrations in the sand. However, in the five locations where Pb exceeded ToA, it showed the highest value in the clay. Cobalt was rather evenly distributed among the fractions. If calculated in relation to the proportion of the particle-size fractions (Table S1 of Supplementary Materials), on average, Cr and Hg of the clay contributed nearly 50% to the soil total concentration, whereas for Ni and Zn both clay and silt contributed for ≈40%. In the case of Co, Cu, and Pb the silt fraction was the main contributor to the total content. Since it is usually expected that the recently added metals concentrate in the clay (Ugwu & Igbokwe, 2019), our observations indicated that a great part of the considered elements has lithogenic or ancient sources.

**Table 5:** Total heavy metals concentration in the clay, silt, and sand obtained with no cement dissolution for the soils from different locations of the city of Ancona (central Italy) and their general mean and median

	Co			Cr			Cu		
	Clay	Silt	Sand	Clay	Silt	Sand	Clay	Silt	Sand
	mg kg^-1^								
*Recreational areas*									
**Altavilla park**	18	23	25	**133**	**68**	**46**	49	53	24
**Cardeto park**	18	19	11	55	31	9	34	39	15
**Cittadella park**	**21**	**21**	**21**	69	31	24	38	34	33
**Palombella park**	13	17	17	34	33	28	**307**	**266**	**276**
Unicef park	20	15	18	98	23	21	46	26	28
Villa Beer park	17	12	35	71	19	17	32	15	27
*Flowerbeds*									
**Cathedral**	18	16	12	39	31	13	56	66	19
**Cavour square**	25	22	18	86	39	19	70	54	34
Corso Carlo Alberto (avenue)	30	19	16	116	44	18	78	51	49
**Harbour, close to the customs house**	23	21	13	**89**	**65**	**41**	**217**	**194**	**77**
**Harbour, close to the Trajan arch**	**25**	**17**	**21**	86	27	58	59	32	31
**Passetto neighbourhood**	**21**	**15**	**31**	**94**	**54**	**16**	58	36	33
**Porta Pia neighbourhood**	23	18	15	78	25	21	72	50	35
**Posatora neighbourhood**	**20**	**17**	**32**	**91**	**31**	**28**	44	27	35
Stamira square	24	17	20	88	24	15	52	33	42
**Torrette neighbourhood**	22	14	31	**107**	**32**	**31**	47	22	23
Viale della Vittoria	24	15	18	102	33	24	59	39	41
*Private gardens*									
**Corso Carlo Alberto **	22	15	15	81	39	15	**155**	**132**	**68**
General mean	21(4)	17(3)	21(7)	84(25)	36(14)	25(13)	82(73)	65(66)	49(59)
Median	22	17	18	87	32	21	57	39	34

	Hg			Ni			Pb			Zn		
	Clay	Silt	Sand	Clay	Silt	Sand	Clay	Silt	Sand	Clay	Silt	Sand
	mg kg^-1^											
*Recreational areas*												
**Altavilla park**	0.20	0.07	0.01	68	47	45	61	74	66	232	173	117
**Cardeto park**	**1.57**	**1.22**	**0.12**	56	51	27	63	63	39	161	178	25
**Cittadella park**	0.46	0.18	0.15	62	40	32	87	91	109	163	107	126
**Palombella park**	0.08	0.02	0.02	34	39	42	75	89	88	**531**	**481**	**153**
Unicef park	0.16	0.16	0.07	97	38	40	49	35	65	143	51	58
Villa Beer park	0.08	0.03	0.01	59	28	60	29	22	39	103	52	63
*Flowerbeds*												
**Cathedral**	**1.88**	**1.14**	**0.35**	47	41	23	**294**	**290**	**74**	455	442	50
**Cavour square**	**1.22**	**0.31**	**0.23**	**98**	**53**	**36**	**208**	**183**	**106**	310	163	107
Corso Carlo Alberto (avenue)	0.09	0.03	0.01	98	43	27	72	61	74	348	241	112
**Harbour, close to the customs house**	**1.55**	**1.33**	**0.31**	**89**	**63**	**38**	**378**	**346**	**274**	**790**	**766**	**396**
**Harbour, close to the Trajan arch**	0.35	0.32	0.07	**115**	**71**	**63**	25	18	83	227	99	97
**Passetto neighbourhood**	0.22	0.12	0.11	**85**	**51**	**46**	59	38	129	275	145	152
**Porta Pia neighbourhood**	**0.61**	**0.43**	**0.13**	70	37	24	**232**	**206**	**228**	345	226	171
**Posatora neighbourhood**	0.08	0.02	0.02	**74**	**39**	**48**	31	25	56	107	63	58
Stamira square	0.21	0.1	0.04	86	40	39	39	23	137	241	93	168
**Torrette neighbourhood**	0.14	0.04	0.03	75	35	36	40	26	45	128	52	45
Viale della Vittoria	0.17	0.05	0.01	91	42	33	44	37	81	225	137	65
*Private gardens*												
**Corso Carlo Alberto **	0.38	0.22	0.03	71	48	32	**177**	**151**	**141**	**785**	**431**	**219**
General mean	0.53(0.59)	0.32(0.44)	0.10(0.11)	76(20)	45(10)	38(11)	109(104)	99(98)	102(63)	309(209)	217(193)	121(87)
Median	0.22	0.14	0.06	75	42	37	62	62	82	237	154	110

Numbers in parentheses are the standard deviations (*n* = 18)

Locations and values in bold refer to those whose soil total content of heavy metals overcame the threshold of attention for each heavy metal (see Table 4)

Clay =  < 2 µm fraction; silt = 2–50 µm fraction; sand =  > 50 µm fraction

The amounts of extractable Cr were always below the detection limit of the method (25 µg kg^−1^), while the other heavy metals showed the following order of extraction: Zn > Pb > Cu > Ni (Table 6), and the general percentage of extractable relative to the total element content ranged from ≈0% for Cr to ≈28% for Pb (Table 6). The highest percentages of extractable Cu and Pb were found in the flowerbed of Viale della Vittoria, whose soil was replaced two years before the sampling; the highest proportion of extractable Ni was obtained for the recreational area of the Altavilla park, and that of Zn for the flowerbed of Stamira square.

**Table 6:** Concentrations of extractable heavy metals and percentage of extractable over the total amount of heavy metals (EXT/TOT) for the soils from different locations of the city of Ancona (central Italy) and their general mean and median

	Cr		Cu		Ni		Pb		Zn	
	Extractable	EXT/TOT	Extractable	EXT/TOT	Extractable	EXT/TOT	Extractable	EXT/TOT	Extractable	EXT/TOT
	mg kg^−1^	%	mg kg^−1^	%	mg kg^−1^	%	mg kg^−1^	%	mg kg^−1^	%
*Recreational areas*										
Altavilla park	bdl	≈ 0	4	8.3	6	12.2	15	22.7	14	7.5
Cardeto park	bdl	≈ 0	3	10.7	3	6.7	14	26.9	35	33.3
Cittadella park	bdl	≈ 0	7	18.9	4	8.0	28	32.2	32	25.8
Palombella park	bdl	≈ 0	30	10.6	3	7.5	11	12.4	40	14.3
Unicef park	bdl	≈ 0	6	18.2	4	7.5	15	35.7	9	11.8
Villa Beer park	bdl	≈ 0	1	4.8	3	7.0	7	28.0	4	6.0
*Flowerbeds*										
Cathedral	bdl	≈ 0	9	22.0	3	9.7	69	35.9	92	34.3
Cavour square	bdl	≈ 0	13	23.2	4	6.8	54	31.2	38	19.9
Corso Carlo Alberto (avenue)	bdl	≈ 0	4	7.0	3	6.0	8	11.4	22	10.4
Harbour, close to the customs house	bdl	≈ 0	30	21.3	3	5.2	43	13.6	17	2.9
Harbour, close to the Trajan arch	bdl	≈ 0	9	25.0	3	3.9	17	37.0	30	25.4
Passetto neighbourhood	bdl	≈ 0	7	16.7	4	6.9	20	32.3	39	22.2
Porta Pia neighbourhood	bdl	≈ 0	18	32.7	5	11.1	44	20.8	87	34.1
Posatora neighbourhood	bdl	≈ 0	2	5.7	3	5.4	10	30.3	4	4.8
Stamira square	bdl	≈ 0	10	26.3	5	9.4	12	27.9	58	41.1
Torrette neighbourhood	bdl	≈ 0	5	16.1	3	5.9	10	32.3	5	6.7
Viale della Vittoria	bdl	≈ 0	18	42.9	4	8.0	19	41.3	47	34.3
*Private gardens*										
Corso Carlo Alberto	bdl	≈ 0	37	29.1	4	8.3	46	28.9	60	12.1
General mean	bdl	≈ 0(-)	12(11)	18.9(10.2)	4(1)	7.5(2.1)	25(18)	27.8(8.6)	35(26)	20.1(11.7)
Median	–	≈ 0	8	18.6	4	7.2	16	29.6	34	19.0
bdl = below the detection limit										

Numbers in parentheses are the standard deviations (*n* = 18)

### Soil biological and biochemical properties

The C_mic_ content was the lowest in the recreational area of Palombella park, and the highest in the flowerbed of Porta Pia (Table 7). For this latter location, we also obtained the lowest amount of respired CO_2_ (399 µg CO_2_–C g^−1^ soil). Because of the combination of the highest C_mic_ content and the lowest ∑CO_2_–C, the flowerbed of Porta Pia showed the lowest qCO_2_ value (0.6 µg CO_2_–C mg^−1^ C_mic_ h^−1^). For all the soils, we obtained a general qCO_2_ mean value of 3.7, with a maximum value of 10.5 at Palombella park. This location showed also the lowest values of the four enzyme activities (Table 7), while the flowerbed of Porta Pia displayed the highest values of alkaline and acid phosphatase activities. The highest ß-glucosidase and urease activities were measured for the Cathedral flowerbed.

**Table 7:** Microbial biomass C content (C_mic_), cumulative amount of CO_2_ evolved during 21 days of incubation (∑CO_2_–C), metabolic quotient (qCO_2_), and activities of alkaline phosphatase, acid phosphatase, ß-glucosidase, and urease for the soils from different locations of the city of Ancona (central Italy) and their general mean and median

	C_mic_	∑CO_2_–C	qCO_2_	Alkaline phosphatase	Acid phosphatase	ß-glucosidase	Urease
	µg C g^−1^ soil	µg CO_2_–C g^−1^ soil	µg CO_2_–C mg^−1^ C_mic_ h^−1^	µg *p*-NP g^−1^ h^−1^			µmol urease g^−1^ h^−1^
*Recreational areas*							
Altavilla park	328	577	3.5	13 874	623	9569	215
Cardeto park	228	644	5.6	11 067	401	5136	102
Cittadella park	626	466	1.5	10 408	362	3313	52
Palombella park	96	508	10.5	6330	77	1229	52
Unicef park	473	489	2.1	13 759	618	9835	223
Villa Beer park	136	474	6.9	12 231	429	4683	98
*Flowerbeds*							
Cathedral	810	514	1.3	15 578	780	10 884	337
Cavour square	428	605	2.8	12 106	513	7461	188
Corso Carlo Alberto (avenue)	139	474	6.8	9668	412	3525	114
Harbour, close to the customs house	308	566	3.6	12 004	540	nd	nd
Harbour, close to the Trajan arch	nd	nd	nd	nd	nd	nd	nd
Passetto neighbourhood	529	938	3.5	17 780	684	8725	285
Porta Pia neighbourhood	1229	399	0.6	19 840	1703	4306	92
Posatora neighbourhood	488	453	1.8	14 117	715	8907	256
Stamira square	nd	nd	nd	nd	nd	nd	nd
Torrette neighbourhood	450	502	2.2	13 438	770	9320	282
Viale della Vittoria	416	744	3.5	12 353	671	6964	257
Private gardens							
Corso Carlo Alberto	259	458	3.5	11 693	342	7012	197
General mean	434(285)	551(134)	3.7(2.6)	12 890(3156)	603(348)	6725(2877)	183(92)
Median	422	505	3.5	12 292	579	7012	197

Numbers in parentheses are the standard deviations (*n* = 16 for C_mic_, ∑CO_2_–C, qCO_2_, alkaline phosphatase, and acid phosphatase; *n* = 15 for ß-glucosidase and urease)

nd = Not determined

## Discussion

### Physicochemical properties of the city soils in comparison with the surrounding soils

The pH range of the urban soils (7.1–7.6) was slightly lower than that of the cultivated soils surrounding the city (Table S2 of Supplementary Materials). This indicates that, rather than to be added of municipal demolition waste, a common practice in urban context that has an alkalinizing effect (Alexandrovskaya & Alexandrovskiy, 2000; Biasioli et al., 2006), the Ancona’s urban soils were added of materials that slightly reduced alkalinity; NH_4_-based fertilizers and organic amendments could have been responsible for this. All the soils showed a large content of clay-size particles. This could raise some concern since soils with fine texture may represent a repository of inorganic and organic particles (partly originated by anthropogenic activities) contributing through wind blasts to increase air levels of PM2.5, which are easily inhaled by humans with deep penetration into the lungs (e.g. Atzei et al., 2019; Unda-Calvo et al., 2019; Wu et al., 2007). The soil textures of the Ancona urban soils appeared to be mostly inherited from the parental material of this area, represented by carbonaceous marine and fluvio-marine sediments, and are similar to those of the rural soils surrounding the city (Table S2 of Supplementary Materials). However, since the substances most involved in heavy metals (selective) adsorption are colloids like clay minerals belonging to the smectites and vermiculites groups (e.g. Gupta & Bhattacharyya, 2012; Malandrino et al., 2006; Otunola & Ololade, 2020), Fe-, Al-, and Mn-oxyhydroxides (e.g. Fialova 2014; Ni et al., 2009; Shi et al., 2021), and organic matter (e.g. Bradl, 2004; Kwiatkowska-Malina, 2018), the high soil clay content can be considered as a positive factor being able to reduce the bioavailability of heavy metals. Since the wind-blowing of the soil fine fraction may contain colloids with adsorbed metals, a proper management of the urban soils aimed at avoiding wind erosion can help reducing the possibility for humans to assimilate heavy metals by inhalation or ingestion (Abrahams, 2002; Higgs et al., 1999; Lasat, 2001; Padoan et al., 2017). Also the presence of active carbonate is a positive aspect because of its capability to induce precipitation/immobilization of many heavy metals (e.g. Huang et al., 2016; Wuana & Okieimen, 2011). The contents of TOC and total N were fairly higher with respect to the highest contents of the rural soils of the surroundings (Table S2 of Supplementary Materials) and were ascribed to the addition of fertilizers and organic amendments. The alteration of the applied organic amendments, with production of acidity, was probably the reason of the generalized lower pH of the urban soils. In addition, other than complexing heavy metals, organic matter is also involved in the formation of soil structure, so creating aggregates sufficiently heavy to reduce wind-blowing. In the case of parks, the soil appeared to be the remainders of in situ soils after they were scalped, reworked, or deeply ploughed. Therefore, with respect to the rural soils of the surroundings, the urban soils showed similar physicochemical and mineralogical properties (Tables S2 and S3 of Supplementary Materials), with higher contents of TOC, total *N*, and available *P* because of the addition of fertilizers and amendments used for green maintenance. Hence, it appears that the urban soils were not massively added of allochthonous earthy materials, unless these latter came from the surroundings. However, in three flowerbeds (two at the Harbour and that of Viale della Vittoria) small amounts of allochthonous materials containing pyroxenes were probably added.

### 4.2 Total concentration and distribution of heavy metals among the particle-size fractions

ToA takes into consideration the mean condition of the city soils, where especially Pb and Zn have been accumulated with time. Then, ToA has also allowed us individuating nine soils where a multi-elemental accumulation occurred. In all the soils where heavy metals exceeded *ToA*, they were more abundant in the clay and, in many cases, in the silt too. This is in agreement with many authors (e.g. Acosta et al., 2009; Ajmone-Marsan et al., 2008; Gong et al., 2014; Liu et al., 2018; Yutong et al., 2016), who found that in the urban soils the fraction < 50 µm was the most involved in heavy metal pollution. This fact farther increases the risk that heavy metals can be inhaled by people (e.g. Acosta et al., 2009; Sah et al., 2019). However, in the soils where Pb did not exceed its *ToA*, sand was the Pb richest fraction. Since Pb is a frequent isomorphic substitute of Ca in carbonaceous rock forming environments (e.g. Cheng et al., 2018; Haldar & Tišljar, 2014; Kumpiene et al., 2008) and these soils contain calcite, we ascribed the relative abundance of Pb in the sand fraction to lithogenic origin (e.g. Acosta et al., 2009; Ajmone-Marsan et al., 2008). For the other six heavy metals, their accumulation in the silt and clay fractions was attributed to their adsorption on clay minerals and Fe-, Al-, and Mn-oxyhydroxides, and to the formation of complexes with humic substances (e.g. Dube et al., 2001; Groenenberg & Lofts, 2014; Peng et al., 2018).

Among the nine soils with two or more metals exceeding the proposed ToA, in the flowerbed close to the custom house at the Harbour and in all the others with Pb over ToA, clay (and often silt) showed the highest Pb contents, indicating that this heavy metal had an additional source other than lithology, probably represented by the past traffic of motor vehicles. The same is probably true for Zn, since railway tracks, services, and junctions have been recognized to be Zn sources for the soils in the vicinity of these facilities in many parts of the World (e.g. Adamu et al., 2017; Akoto et al., 2008; Wiłkomirski et al., 2011; Zhang et al., 2012). For the other four heavy metals (Cr, Cu, Ni, and Hg), being sources like mining, industrial activities, and use of fertilizers and pesticides (e.g. Manta et al., 2002; Rodrigues et al., 2006; Wei & Yang, 2010; Xueqiu et al., 2020) impossible to invoke for the city of Ancona, we suspect they came from the strong trade activities, fishery, the movement of more than 1.5 million tourists the harbour experiences yearly, but also from metallurgy, construction, and other human activities that succeeded since the foundation of the city.

In the four locations affected by high content of three heavy metals, when contaminants are represented by Pb and Zn, since we did not take into consideration atmospheric deposition and irrigation water as possible sources, their excessive content was ascribed to the already mentioned vehicular traffic and railway, respectively. Thus, particular is the case of the flowerbeds of Passetto and Posatora, which are at the extreme east and west of the city, respectively (≈4 km of distance one from the other) but were contaminated by the same heavy metals: Co, Cr, and Ni. A certain amount of Cr and Ni could come from antigorite, which formed 6% of Passetto and 1% of Posatora soils. The antigorite in the Passetto soil contained 632 ± 21 mg kg^−1^ Cr and 711 ± 16 mg kg^−1^ Ni, while that in the Posatora soil comprised 553 ± 23 mg kg^−1^ Cr and 634 ± 24 mg kg^−1^ Ni. By considering the antigorite content, the amounts of Cr and Ni of mineral origin accounted for ≈38 mg kg^−1^ Cr and 43 mg kg^−1^ Ni in the Passetto soil, and for ≈6 mg kg^−1^ Cr and Ni in the Posatora soil. Regarding the source of Cr and Ni exceeding the antigorite contribution in these two, but also in the other investigated soils depending on the relative antigorite content (ranging from ≈2.5 to ≈31 mg kg^−1^ for Cr and from ≈4 to ≈34 mg kg^−1^ for Ni), we do not offer explanation except for the diffuse source of pollution typical of historical cities where all human activities succeeded with time (e.g. Christoforidis & Stamatis, 2009; Luo et al., 2015; Tume et al., 2018).

Among the locations with two heavy metals overcoming ToA, particular is the case of the Palombella park, which resulted contaminated by Cu and Zn, with the highest content of total Cu (282 mg kg^−1^). This park is close to a nowadays dismissed brick kiln, and this type of industrial activity has been assessed to be the source of heavy metals like Cu, Pb, and Zn that accumulate in the soils surrounding the factory (e.g. Achakzai et al., 2015; Begum et al., 2015; Bisht & Neupane, 2015; Ishaq et al., 2010; Ismail et al., 2012). Because of this, we attributed most of the contents of Cu and Zn found in the soil of Palombella park to the activity of the dismissed brick kiln. For all the other situations with one of more heavy metals overcoming ToA, we considered the relatively high level of contamination was due to all human activities that in cities funded century or millennia ago may leave trace into the soil, or to accidental contaminations typical of cities with strong trade activities combined with vehicular, railway, and naval traffic. This, in turn, represents the serious problem to define a fixed reference threshold for historical cities.

### Extractability of heavy metals in the city soils

Soil functionalities are compromised when the availability of heavy metals is sufficiently high to interfere with soil biological activities (e.g. Khan et al., 2007; Wang et al., 2018; Xian et al., 2015). Because of this, many authors have determined the availability of heavy metals to assess the thresholds over which soil functions might be compromised (e.g. Basta et al., 2005; Madrid et al. 2008; Yutong et al., 2016). In the soils of Ancona, the percentage of availability over the total content of heavy metal followed the order Pb > Zn≈Cu > Ni > Cr. In general, when soil has sub-alkaline pH, the chemical availability of heavy metals is small and, when they are introduced into the soil or released by weathering of the mineral lattices, they are immobilized as hydroxides or co-precipitate with secondary carbonates (e.g. Huang et al., 2016; Kabata-Pendias & Pendias, 2001). Then, when in cationic form, heavy metals can be adsorbed by clay minerals and carbonates, or complexed by humic substances, especially at sub-alkaline pH (e.g. Chuan et al., 1996; Peganova & Edler, 2004), so slowing down their availability. However, the amount of extractable Cu, Ni, Pb, and Zn of our soils gave not significant tendency lines when related to total carbonates, active carbonate, TOC, and clay minerals (2:1 clay minerals plus kaolinite) content (Fig. S1 of Supplementary Materials). Therefore, the absence of reliable relationships among the extractable content of heavy metals and the main soil properties usually involved in their immobilization was ascribed to two factors: (*i*) the weathering rate of the minerals comprising them, and (*ii*) at least for the unknown quote due to contamination, the chemical form in which the heavy metals arrived to the soil and the chemical reactions to which they are submitted, given the soil geochemical conditions like pH, moisture, redox conditions, presence of complexing substances. For example, the availability of Cr and Ni present in antigorite depends on the weathering rate of the crystal lattice that, at sub-alkaline pH, is expected to be low. Instead, for Cu, Pb, and Zn, the extractable portion probably derived from recent anthropic additions in forms that were not immobilized through the redistribution among the main chemical/mineralogical phases, yet. The fact that Pb showed the highest proportion of extractability and abounded in the coarser separates was ascribed to the relatively high solubility of Pb-hydroxides in sub-alkaline soils, as reported by Basta et al. (2005) and Chuan et al. (1996).

### Biological and biochemical properties of the city soils

Besides detecting the amount of extractable heavy metals, it is important to assess if the levels of these elements may have negatively affected soil biology, so compromising soil functions. As extensively reported in the literature (e.g. Bastida et al., 2008; Schloter et al., 2003; da Silva Aragão et al., 2020), parameters like C biomass, basal respiration, qCO_2_, and enzyme activities are widely considered as proxies for soil quality as they are intimately linked to physicochemical parameters able to influence soil biological activity (Dantas Lopes et al., 2021; Khan et al., 2016; Lorenz & Kandeler, 2005). Although basal respiration is not always considered a reliable indicator for heavy metal polluted soils (Romero-Freire et al., 2016), the mean ∑CO_2_–C of 551 µg CO_2_–C g^−1^ for these urban soils was higher than the values found in the superficial horizon of vineyard soils (from 66.6 to 157.5 µg CO_2_–C g^−1^ soil, Corti et al., 2007) and forest soils (434 µg CO_2_–C g^−1^ soil, Cocco et al., 2013), located close to the city. Similarly, Decina et al. (2016) found that soil respiration rates decreased from urban soils to rural forest soils in the Boston metropolitan area and ascribed this trend to the soil management in the various situations, which are variably able to stimulate the microbial activity. The amount of CO_2_–C respired per unit of C_mic_ and time of respiration gives the qCO_2_ index of microbial activity linked to the soil environmental conditions, and relatively low qCO_2_ values indicate microbial adaptation to environmental soil conditions (e.g. Anderson & Domsch, 1990, 1993; Hannachi et al., 2014; Moscatelli et al., 2007). The relatively low qCO_2_ values of the studied soils, with respect to ≈28 µg CO_2_–C g^−1^ assessed in city surrounding soils (Corti et al., 2007), indicated the high substrate use efficiency of the microbial community harbouring these soils (Anderson & Domsch, 1989), which means a prevalence of anabolic over catabolic processes (Chander & Brookes, 1991) promoted by relatively well-adapted microbial communities (Hannachi et al., 2014). Therefore, if more carbon is available for biomass production, a higher proportion of C_mic_ to TOC should occur (Anderson & Domsch, 1986). In fact, while urban soils gave a mean C_mic_/TOC proportion of ≈1.3%, it was ≈0.1% in the vineyard soils (Corti et al., 2007) and ≈0.5% in the forest soils (Cocco et al., 2013) close to the city. The high C use efficiency of the microbial communities harbouring the urban soils was probably due to their sub-alkaline pHs, as acid conditions and nutrient limitations produce a reduction of microbial C use efficiency (Jones et al., 2019; Keiblinger et al., 2010; Sinsabaugh et al., 2013). However, it also means that soil microbial functions were not compromised by the contamination level reached by these soils in terms of heavy metals or other pollutants. As a further demonstration of this, the flowerbed of Porta Pia displayed the highest contents of available Ni and Zn and the highest C_mic_ content; in contrast, the flowerbed of Corso Carlo Alberto and the Palombella and Villa Beer parks showed the lowest C_mic_ concentration and not excessive extractable heavy metals, except for Cu at Palombella park.

Alkaline phosphatase activity was higher than acid phosphatase activity because of the sub-alkaline soil pH (Dick et al., 2000; Marinari & Vittori Antisari, 2010; Zhan & Sun, 2014). Enzymes like phosphatase, β-glucosidase, and urease are stabilized by humic and/or clay colloids and preserve much of their activity (Busto & Perez-Mateos, 2000; Gianfreda et al., 1995; Hayano & Katami, 1977; Pająk et al., 2016; Rao et al., 2000). By comparing our data with those reported by Nannipieri et al. (2002), it appeared that the general enzyme activity of our soils was not depressed, except for the soil at Palombella park, where enzyme activities and C_mic_ were the lowest, and the qCO_2_ the highest. For this soil, for decades close to a brick kiln, it is possible that it received elevated amounts of Cu and Zn, whose availability was not efficiently reduced notwithstanding the sub-alkaline pH; the low clay content was probably responsible for this (Aponte et al., 2020). Thus, we attribute the depressed enzyme activities and microbial conditions to the relatively high content of both available Cu and Zn and the lowest clay content. However, studies have found opposite correlations between enzyme activities and heavy metal contents (e.g. Aponte et al., 2020; Tang et al., 2020), probably because of the different analytical methods applied, pollution levels, soil properties, etc.

## Conclusions

Many countries adopted regulatory guidelines establishing limits for the total content of metal(loid)s in the soil, but these guidelines do not differentiate soils of urban areas from soils earmarked for other uses. Thus, especially for urban soils of old-settled cities where metallurgy, construction, and other human activities have accumulated heavy metals in soil for centuries or millennia, the adoption of fixed limits for heavy metals concentration could make difficult to respect them. The proposed new index, ToA, has the advantage to represent a heavy metal risk assessment calibrated on the base of city material history and is a mobile limit that is subjected to decrease or increase depending on the care given to the urban soils. We tested this new index in the urban soils of an old-settled city like Ancona (Italy) and found that for five out of the seven considered elements (Co, Cr, Cu, Hg, and Ni) ToA was lower, while for Pb and Zn it was higher than the limits reported by the Italian legislation. The proposed ToA allowed us recognizing nine over 18 soils with a multi-elemental critical situation and was therefore considered a valuable tool to highlight soils where it is more impellent to intervene to mitigate or avoid potential environmental concerns in old-settled cities. However, despite the total and available concentration of heavy metals in soil, the soil biological functions (microbial and enzyme activities) were maintained in most of the 18 situations, as well as soil ecological services (e.g. substrate for plant growth, organic carbon storage, water filtration, chemical and biochemical transformation) were ensured. Because of this, for a proper risk assessment in urban soils, we suggest considering both the content of available heavy metals and the conservation of soil functions and ecological services. However, we are aware that more research is needed before ToA or other risk assessment tools based on available heavy metal content could be adopted as regulatory limit.

## Supplementary Information

Below is the link to the electronic supplementary material.

Supplementary file1 (DOCX 229 KB)

## Data Availability

The datasets generated and/or analysed during the current study are available from the corresponding author on reasonable request.
