# Supplementary material for: Heavy metal load and effects on biochemical properties in urban soils of a medium-sized city, Ancona, Italy
Source: Environ Geochem Health. 2021 Oct 6;44(10):3425–49. doi: 10.1007/s10653-021-01105-8 (PMC9522685; doi:10.1007/s10653-021-01105-8)
Supplement: Supplementary file 1 — Supplementary file1 (DOCX 229 KB) [file 10653_2021_1105_MOESM1_ESM.docx]

**Heavy metal load and effects on biochemical properties in urban soils of a medium-sized city, Ancona, Italy**

Dominique Serrani^a*^, Franco Ajmone-Marsan^b^, Giuseppe Corti^a^, Stefania Cocco^a^, Valeria Cardelli^a^, Paola Adamo^c^

^a^Department of Agriculture, Food and Environmental Sciences, Polytechnic University of Marche, Via Brecce Bianche 10, 60131 Ancona, Italy

^b^Department of Agriculture, Forest and Food Sciences, University of Turin, Largo Paolo Braccini 2, 10095 Grugliasco, Italy

^c^Department of Agricultural Sciences, University of Naples Federico II, Via Università 100, 80055 Portici, Italy

*Corresponding author at: Department of Agriculture, Food and Environmental Sciences, Polytechnic University of Marche, Via Brecce Bianche 10, 60131 Ancona (AN), Italy

*Email address:* d.serrani@univpm.it (D. Serrani)

SUPPLEMENTARY MATERIALS

**Point 1. Details on the selected locations**

For the six locations inside recreational areas, soil samples were collected in the surrounding of children’s play areas, where higher is the risk for human health.

For the flowerbeds, we excluded those made by reconstructed soil resting on impermeable surface (asphalt or concrete) and selected those in contact with the *in-situ* soil, even though we could not exclude disturbances like additions of allochthonous materials. The selected 11 flowerbeds had different surfaces and represented different situations within the city; in all these cases, the soil samples were collected in the middle of the flowerbed. The Municipality service adopts a management guideline consisting in tree pruning for the parks, and flower changing/maintenance for the flowerbeds. On renovation, that may happen yearly for herbaceous flowers or every 3-10 years for bushy flowers, the soil is gently hoed and small amounts of fertilizers and/or amendments are applied. The use of herbicides is not allowed, while pesticides have occasionally been distributed to fight pests attacking historical trees.

A location with private gardens with an ongoing cultivation of vegetables, flowers, and few bushes was also selected.

| Table S1. Percentage contribution of clay, silt, and sand to the total content of heavy metals for the soils from different locations of the city of Ancona (central Italy), and the general mean referred to each separate. Locations in bold are those with one or more heavy metals overcoming the threshold of attention. | | | | | | | | | | | | | | | | | | | | | |
| --- | --- | --- | --- | --- | --- | --- | --- | --- | --- | --- | --- | --- | --- | --- | --- | --- | --- | --- | --- | --- | --- |
|  | Co | | | Cr | | | Cu | | | Hg | | | Ni | | | Pb | | | Zn | | |
|  | Clay | Silt | Sand | Clay | Silt | Sand | Clay | Silt | Sand | Clay | Silt | Sand | Clay | Silt | Sand | Clay | Silt | Sand | Clay | Silt | Sand |
|  | % | | | | | | | | | | | | | | | | | | | | |
| *Recreational areas* | | | | | | | | | | | | | | | | | | | | | |
| **Altavilla park** | 26 | 53 | 21 | 50 | 40 | 10 | 34 | 57 | 9 | 63 | 35 | 2 | 41 | 44 | 15 | 29 | 54 | 17 | 41 | 48 | 12 |
| **Cardeto park** | 28 | 42 | 30 | 48 | 38 | 14 | 29 | 48 | 23 | 45 | 49 | 6 | 32 | 41 | 27 | 29 | 40 | 31 | 35 | 55 | 10 |
| **Cittadella park** | 33 | 58 | 9 | 53 | 42 | 5 | 36 | 56 | 8 | 56 | 39 | 5 | 44 | 50 | 6 | 31 | 58 | 11 | 42 | 49 | 9 |
| **Palombella park** | 10 | 23 | 67 | 15 | 24 | 61 | 14 | 21 | 65 | 37 | 16 | 47 | 11 | 21 | 68 | 11 | 23 | 66 | 25 | 39 | 36 |
| Unicef park | 36 | 47 | 17 | 66 | 27 | 7 | 44 | 42 | 14 | 34 | 58 | 8 | 53 | 36 | 11 | 34 | 42 | 24 | 55 | 34 | 12 |
| Villa Beer park | 32 | 32 | 35 | 66 | 25 | 8 | 47 | 31 | 21 | 63 | 33 | 4 | 45 | 30 | 24 | 36 | 38 | 26 | 49 | 35 | 16 |
| *Flowerbeds* | | | | | | | | | | | | | | | | | | | | | |
| **Cathedral** | 20 | 44 | 36 | 26 | 51 | 23 | 20 | 60 | 19 | 33 | 50 | 17 | 22 | 48 | 30 | 24 | 59 | 17 | 27 | 65 | 8 |
| **Cavour square** | 29 | 51 | 20 | 47 | 43 | 10 | 33 | 52 | 15 | 59 | 31 | 11 | 41 | 45 | 14 | 30 | 55 | 15 | 42 | 45 | 14 |
| Corso Carlo Alberto (avenue) | 30 | 36 | 34 | 49 | 35 | 16 | 28 | 35 | 37 | 54 | 34 | 13 | 41 | 35 | 24 | 21 | 34 | 45 | 33 | 44 | 23 |
| **Harbour, close to the customs house** | 25 | 37 | 37 | 29 | 35 | 36 | 29 | 43 | 28 | 34 | 48 | 18 | 31 | 35 | 34 | 23 | 34 | 43 | 26 | 41 | 34 |
| **Harbour, close to the Trajan arch** | 22 | 29 | 49 | 30 | 18 | 52 | 29 | 31 | 40 | 30 | 54 | 16 | 28 | 33 | 39 | 9 | 13 | 78 | 34 | 29 | 38 |
| **Passetto neighbourhood** | 26 | 40 | 34 | 42 | 51 | 6 | 36 | 47 | 18 | 39 | 45 | 17 | 36 | 46 | 17 | 23 | 32 | 45 | 38 | 43 | 19 |
| **Porta Pia neighbourhood** | 35 | 49 | 16 | 57 | 32 | 11 | 39 | 48 | 13 | 42 | 52 | 6 | 46 | 43 | 11 | 31 | 48 | 21 | 40 | 46 | 14 |
| **Posatora neighbourhood** | 37 | 39 | 24 | 65 | 27 | 8 | 48 | 37 | 15 | 71 | 22 | 7 | 52 | 34 | 13 | 37 | 37 | 26 | 51 | 37 | 11 |
| Stamira square | 33 | 50 | 17 | 60 | 34 | 6 | 35 | 48 | 17 | 47 | 47 | 5 | 44 | 44 | 12 | 23 | 29 | 48 | 45 | 37 | 19 |
| **Torrette neighbourhood** | 41 | 37 | 21 | 65 | 28 | 7 | 54 | 36 | 10 | 67 | 27 | 5 | 55 | 36 | 9 | 43 | 40 | 17 | 59 | 34 | 7 |
| Viale della Vittoria | 34 | 41 | 25 | 54 | 33 | 12 | 34 | 43 | 23 | 62 | 34 | 4 | 45 | 39 | 16 | 23 | 36 | 41 | 41 | 47 | 11 |
| *Private gardens* | | | | | | | | | | | | | | | | | | | | | |
| **Corso Carlo Alberto** | 39 | 43 | 18 | 52 | 41 | 7 | 37 | 52 | 11 | 50 | 47 | 3 | 41 | 46 | 13 | 34 | 47 | 19 | 48 | 43 | 9 |
| General mean | 30(8) | 42(9) | 28(14) | 49(15) | 35(9) | 17(16) | 35(10) | 44(10) | 21(14) | 49(13) | 40(12) | 11(10) | 39(11) | 39(7) | 21(15) | 27(9) | 40(12) | 33(19) | 41(10) | 43(9) | 17(10) |
| Numbers in parentheses are the standard deviations for general mean (*n* = 18). | | | | | | | | | | | | | | | | | | | | | |

| Table S2. Physicochemical and biological properties of the A horizons from the surrounding soils of Ancona (from the literature). | | | | | |
| --- | --- | --- | --- | --- | --- |
|  | pH | Total organic C | Total N | Available P | |
|  |  | g kg^-1^ | | mg kg^-1^ |  |
| Corti et al. (2006), cultivated soils | /* | 5.2 – 9.4 | /* | /* | |
| Corti et al. (2007), cultivated soils | ≈ 8.3 | ≈ 8.15 | ≈ 0.32 | ≈ 2 | |
| Corti et al. (2011), cultivated soils | 7.7 - 8.0 | 11.5 – 22.1 | 1.8 – 2.0 | 0.5 – 6.1 | |
| Corti et al. (2019), forest soils | 6.19 – 7.23 | 24.1 – 102.2 | 2.0 – 7.9 | 6.2 – 25.1 | |

*data not available in the reference.

| Table S3. Mineralogical composition (semi-quantitative estimation) of the A horizons from the surrounding soils of Ancona (from the literature). | | | | | | | | | | | | | | | |
| --- | --- | --- | --- | --- | --- | --- | --- | --- | --- | --- | --- | --- | --- | --- | --- |
|  | Q | P | C | G | D | M | Ch | K | 2:1 CM | HIV | HIV/HIS | M-S | S | V | OM |
|  | % | | | | | | | | | | | | | | |
| Corti et al. (2011), cultivated soils | 10 - 20 | 15 - 20 | 30 - 35 | / | 5 | 5 - 10 | 5 - 10 | 5 | 10 - 15 | / | / | / | / | / | / |
| Brecciaroli et al. (2012), cultivated soils | 16 | 17 | 32 | / | 4 | 6 | 7 | 6 | 12 | / | / | / | / | / | / |
| Cocco et al. (2013), forest soils | 20 - 22 | 10 - 11 | 23 - 24 | tr - 3 | tr - 3 | 8 - 9 | / | 11 - 14 | / | / | 13 -15 | 1 - 3 | 2 - 3 | tr - 2 | 1 - 3 |
| Corti et al. (2019), forest soils | 28 -29 | 24 | / | / | / | 12 - 14 | / | 10 - 12 | / | 7 | 15 - 18 | / | / | / | / |
| Q = quartz; P = plagioclases; C = calcite; G = gypsum; D = dolomite; M = micas; Ch = primary chlorite; K = kaolinite, 2:1 CM = clay minerals with 2:1 structure; HIV = hydroxy-Al interlayered vermiculite; HIV/HIS = mixture of hydroxy-Al interlayered vermiculite (HIV) and hydroxy-Al interlayered smectite (HIS) at various degree of hydroxy-Al polymerization; M–S = interstratified mica–smectite (mostly 2:1); S = smectites; V = vermiculites; OM = other minerals (amphiboles, serpentine, talc).  tr = < 1%. | | | | | | | | | | | | | | | |


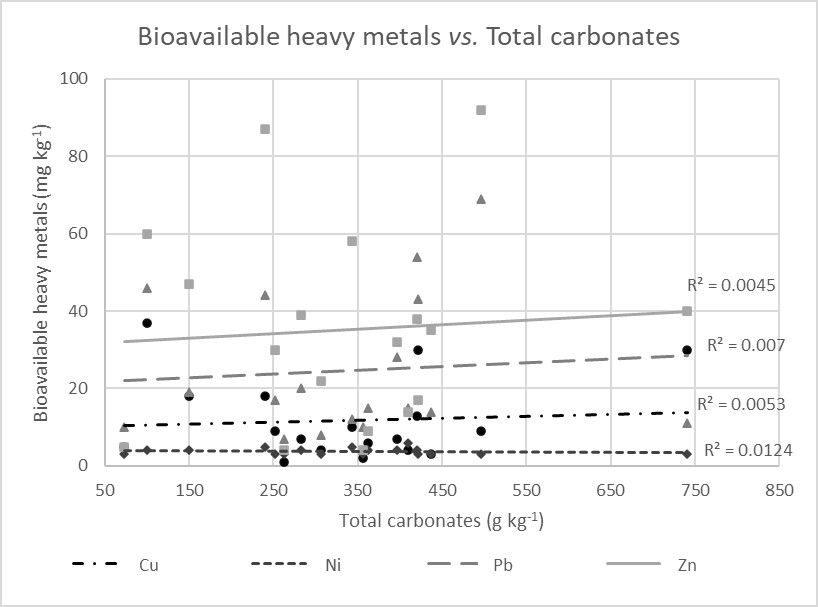

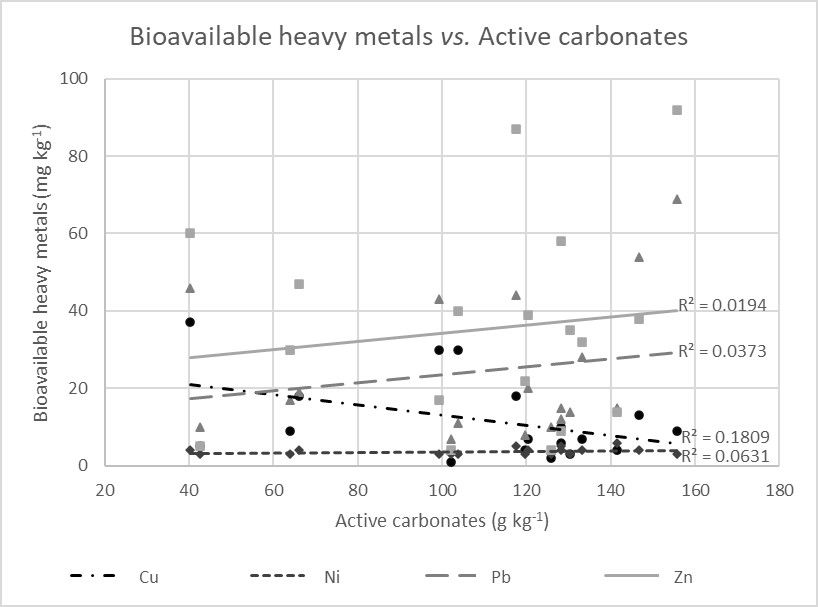


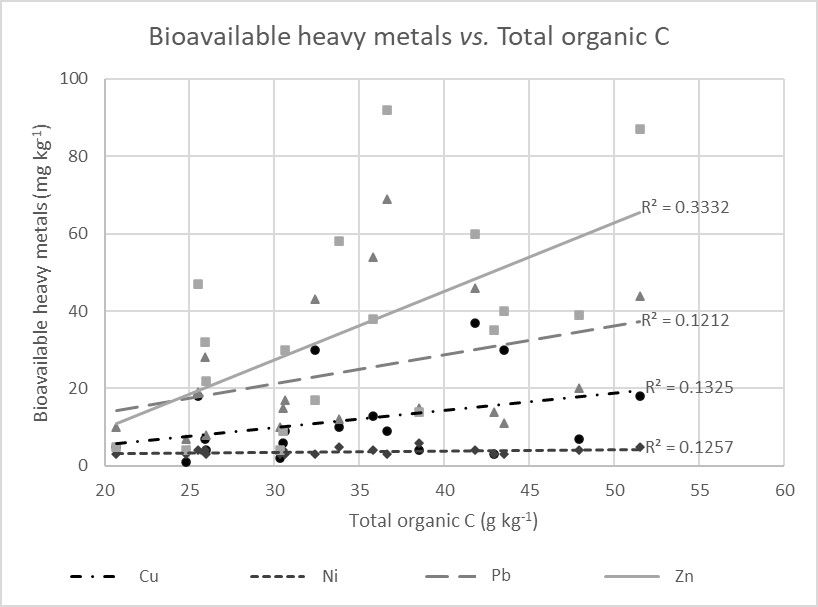


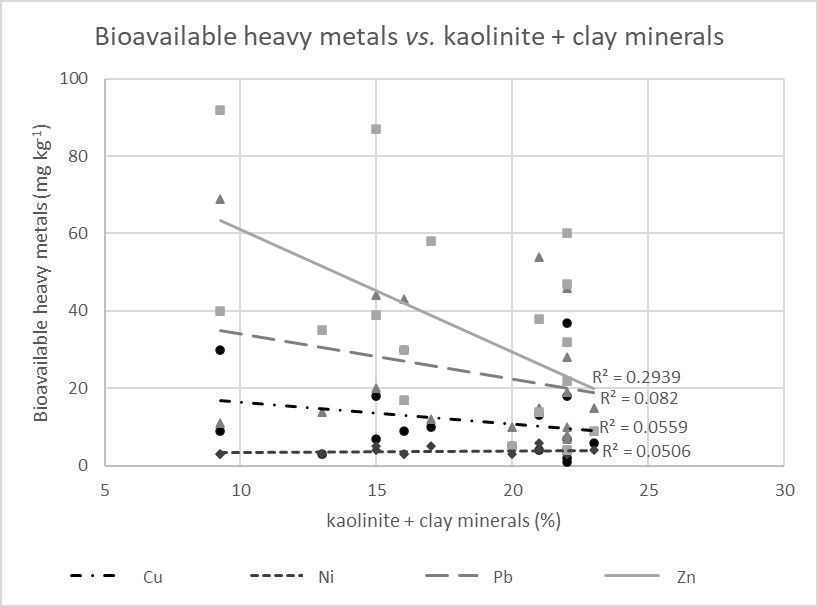


Figure S1. Relations and tendency lines with *R^2^* between the contents of bioavailable heavy metals and total carbonates, active carbonates, total organic carbon (TOC), and kaolinite + clay minerals.

**References**

Brecciaroli, G., Cocco, S., Agnelli, A., Courchesne, F., & Corti, G. (2012). From rainfall to throughfall in a maritime vineyard. *Science of the Total Environment*, 438, 174–188.

Cocco, S., Agnelli, A., Gobran, G. R., & Corti, G. (2013). Changes induced by the roots of *Erica arborea* L. to create a suitable environment in a soil developed from alkaline and fine-texture marine sediments. *Plant and Soil*, 368, 297-313.

Corti, G., Agnelli, A., Cocco, S., Cardelli, V., Masse, J., & Courchesne, F. (2019). Soil affects throughfall and stemflow under Turkey oak (*Quercus cerris* L.). *Geoderma*, 333, 43-56.

Corti, G., Agnelli, A., Cuniglio, R., Cocco, S., & Orsini, R. (2006). Studio pedologico di dettaglio di due microbacini della collina interna marchigiana. In S. Esposito, C. Epifani, C. Serra (Eds) Progetto di Ricerca “CLIMAGRI – Cambiamenti climatici e agricoltura”, Ministero delle Politiche Agrarie, Alimentari e Forestali. Climagri (pubbl. No 51. CRA UCEA, pp. 129-141). Roma.

Corti, G., Cavallo, E., Cocco, S., Biddoccu, M., Brecciaroli, G., & Agnelli, A. (2011). Evaluation of erosion intensity and some of its consequences in vineyards from two hilly environments under a Mediterranean type of climate, Italy, chapter 6. In D. Godone, S. Stanchi (Eds) Soil Erosion in Agriculture. *Tech Open Access Publisher*. Rijeka, Croatia.

Corti, G., Cocco, S., Agnelli, A., & Basili, M. (2007). Suolo bulk e rizosferico in vigneti con differenti gestioni del suolo. *Italus Hortus*, 14, 367-371.
